# Unique Chemokine Profiles of Lung Tissues Distinguish Post-chemotherapeutic Persistent and Chronic Tuberculosis in a Mouse Model

**DOI:** 10.3389/fcimb.2017.00314

**Published:** 2017-07-13

**Authors:** Soomin Park, Seung-Hun Baek, Sang-Nae Cho, Young-Saeng Jang, Ahreum Kim, In-Hong Choi

**Affiliations:** Department of Microbiology, Institute for Immunology and Immunological Diseases, and Brain Korea 21 PLUS Project for Medical Science, Yonsei University College of Medicine Seoul, South Korea

**Keywords:** active tuberculosis, persistent tuberculosis, mouse model, chemokines, lung tissues, cDNA microarrays

## Abstract

There is a substantial need for biomarkers to distinguish latent stage from active *Mycobacterium tuberculosis* infections, for predicting disease progression. To induce the reactivation of tuberculosis, we present a new experimental animal model modified based on the previous model established by our group. In the new model, the reactivation of tuberculosis is induced without administration of immunosuppressive agents, which might disturb immune responses. To identify the immunological status of the persistent and chronic stages, we analyzed immunological genes in lung tissues from mice infected with *M. tuberculosis*. Gene expression was screened using cDNA microarray analysis and confirmed by quantitative RT-PCR. Based on the cDNA microarray results, 11 candidate cytokines genes, which were obviously up-regulated during the chronic stage compared with those during the persistent stage, were selected and clustered into three groups: (1) chemokine genes, except those of monocyte chemoattractant proteins (MCPs; CXCL9, CXCL10, CXCL11, CCL5, CCL19); (2) MCP genes (CCL2, CCL7, CCL8, CCL12); and (3) TNF and IFN-γ genes. Results from the cDNA microarray and quantitative RT-PCR analyses revealed that the mRNA expression of the selected cytokine genes was significantly higher in lung tissues of the chronic stage than of the persistent stage. Three chemokines (CCL5, CCL19, and CXCL9) and three MCPs (CCL7, CCL2, and CCL12) were noticeably increased in the chronic stage compared with the persistent stage by cDNA microarray (*p* < 0.01, except CCL12) or RT-PCR (*p* < 0.01). Therefore, these six significantly increased cytokines in lung tissue from the mouse tuberculosis model might be candidates for biomarkers to distinguish the two disease stages. This information can be combined with already reported potential biomarkers to construct a network of more efficient tuberculosis markers.

## Introduciton

Approximately 5–10% of latent infections of tuberculosis (TB) become active over the lifetimes of infected individuals (Sia and Wieland, 2011). Therefore, the most effective strategy for TB control is the early diagnosis of an active infection in individuals with latent TB followed by preventive chemotherapy (Verhagen et al., 2013; Abouda et al., 2015; Lee et al., 2015b). Currently, *in vitro* studies of blood lymphocyte stimulation with *Mycobacterium tuberculosis* (Mtb)-specific antigens or detection of cytokines in plasma are the most popular assays used for the diagnosis of active infections. The interferon-gamma (IFN-γ) release assay (IGRA) is a highly sensitive and specific method for detecting Mtb infection (Greveson et al., 2013), but it cannot distinguish latent from active infections efficiently (Lange et al., 2012; Targowski et al., 2014; Cho et al., 2016), despite it has been commonly used for the screening and clinical diagnosis of these two stages, respectively.

One method to find more suitable biomarkers of TB is to compare the cytokines in sera from patients with active or latent stages of infection with those from healthy individuals (Weiner and Kaufmann, 2014; Jayakumar et al., 2015; Walter et al., 2016). However, consistent results have not been obtained because the number of cytokines analyzed is often too small, and the types of assays used to detect them vary between studies. Thus, a more systematic approach for analyzing all cytokines is needed, and the results obtained can be combined with reported TB biomarkers to construct a network of potential markers. This study therefore aimed to identify cytokines that could be superior or additively informative to IFN-γ for the rapid diagnosis of TB and for distinguishing between latent and active infections.

Instead of human samples, we analyzed lung tissues from mice infected with Mtb strain Erdman, which was established by our group based on the modified Cornell mouse model for tuberculosis. Because of their heterogeneous genetic background, human samples such as peripheral blood lymphocytes or plasma often show diverse immune responses, and, moreover, may reflect an indirect phenomenon far from the pathological lesions. Therefore, we suggest that an animal study will deliver consistent information of *in situ* immune responses because infected lung tissues from the same genetic background are evaluated. Through cDNA microarray and quantitative RT-PCR analyses, we obtained information on immune response-related genes expressed differentially according to the persistent and chronic stages, and attempted to identify biomarker candidates to distinguish the two infection stages of TB.

## Materials and methods

### Bacterial strains

The infecting strain, Mtb Erdman, was grown in Middlebrook 7H9 broth (Difco, Oxford, UK) supplemented with 0.05% Tween 80 and albumin-dextrose-catalase enrichment.

### Mice infection model

C57BL/6 mice were infected through the aerosol route with Mtb (200–300 CFUs/lung), using a Glas-Col aerosol exposure system. For the chronic stage group, the mice were not treated with anti-TB agents after bacilli infection. For the persistent stage group, the mice were treated with anti-TB agents beginning at 4 weeks post-infection to induce the persistent stage of infection. As the first therapy, the infected mice were treated with INH and PZA for 5 weeks and subsequently with INH and EMB for a further 3 weeks. The drugs were delivered *ad libitum* by adding the following concentrations to drinking water: 100 μg/mL of INH, 600 μg/mL of EMB, and 600 μg/mL of PZA. All drug-containing water was replaced weekly. Water consumption was monitored to determine the delivered daily dose (INH: 26.5 ± 0.9 mg/kg; PZA and EMB: 132.6 ± 4.7 mg/kg). To maintain the persistent stage of infection, mice that had completed the drug therapy were supplied with regular drinking water for 1–2 weeks. A third group of mice not subjected to bacilli infection or drug therapy was used as the healthy controls.

For the reactivation stage, the mice in the persistent stage were supplied with regular drinking water for further 4 weeks. Chronic stage mice were sacrificed at 16 weeks post-infection without any drug therapy. At the indicated time points, 3–4 mice in each group were sacrificed, the lungs and spleens were removed and homogenized in phosphate-buffered saline, and dilutions of the tissue homogenates were plated on 7H10 agar to enumerate the CFUs.

### Histopathology

One lone of the lungs was fixed in 4% phosphate-buffered formaldehyde and embedded in paraffin. Sections of ~5 μm thickness were prepared and stained with haematoxylin and eosin. Acid-fast Mtb bacilli in the lung tissue were detected by Ziehl-Neelsen staining. The stained slides were observed under a light microscope (×40). Lung inflammation lesions were evaluated by ImageJ software (National Institutes of Health, Bethesda, Maryland, USA). Results were represented as the percentage of area with lesions.

### RNA isolation

Total RNA was isolated from lung tissues of all three groups of mice, using the RNeasy Mini Kit (Qiagen, Valencia, CA, USA). The quality and quantity of total RNA were assessed by measuring the absorbance ratios of total RNA at 260/280 and 260/230 nm with the NanoDrop-2000 spectrophotometer (Thermo Fisher Scientific, Wilmington, DE, USA), as well as the 28S/18S ratio and RNA integrity number with the Agilent 2100 BioAnalyzer.

### cDNA synthesis and microarray analysis

For cDNA synthesis, reverse transcription was performed with M-MLV Reverse Transcriptase (Invitrogen, Carlsbad, CA, USA). Each 40 μL reaction included 8 μL of 5 × first-strand buffer, 8 μL of dNTP (2.5 mM), 1 μL of oligo (dT) primer (0.5 μg/μL), 2 μL of Moloney murine leukemia virus reverse transcriptase, 4 μL of dithiothreitol (0.1 M), 3 μg of RNA template, and a suitable volume of RNase-free water for each sample. This mixture was incubated at 42°C for 2 h. For microarray analysis, a GeneChip® (Mouse Gene 2.0 ST Array X 21; Affymetrix, Santa Clara, CA, USA) containing more than 698,000 total probes and 26,515 RefSeq (Entrez) genes was used in this study. Samples were prepared and handled according to the manufacturer's recommendations. Significant transcripts with expression value changes of 2-fold or greater were selected, using a *t*-test with *p* < 0.05.

### Real-time reverse transcription (RT)-PCR

All primers for the DNA oligonucleotides and the housekeeping gene glyceraldehyde-3-phosphate dehydrogenase (GAPDH) were synthesized by the IDT real-time PCR primer design program. Real-time PCR was performed with FastStart Universal Power SYBR Green Master (ROX) (Roche Diagnostics, Indianapolis, IN, USA) using the 7500 Real-Time PCR system (Applied Biosystems, Foster City, CA, USA). In brief, 1 μL of cDNA was added to a PCR mixture consisting of 12.5 μL of FastStart SYBR green master mix, 11 μL of RNase-free water, and 10 μM of each primer (Table S4). The real-time PCR running protocol was 10 min at 95°C, followed by 40 cycles of 95°C for 15 s and 60°C for 1 min. The dissociation curve was obtained by heating from 60 to 95°C. For data analysis, StepOne version 2.0.2 software (Applied Biosystems) was used to calculate the levels of target gene expression in samples relative to the expression level in the control samples, using the comparative cycle threshold method (ΔΔCT). Expression values for target genes were normalized against that of GAPDH.

### Statistical analysis

Results are presented as the mean and standard error of the mean (SEM). For the quantitative RT-PCR analysis, Student's unpaired *t*-test (two-tailed) was used. For analysis of CFU score and inflammation score, one-way ANOVA was used. Data were analyzed using the GraphPad Prism version 5.01 program (GraphPad Software Inc., USA). A *p* < 0.05 was considered statistically significant.

## Results

### Colony-forming unit counts and histopathological findings in lung tissues from mice infected with Mtb bacilli

**Figure 1:**
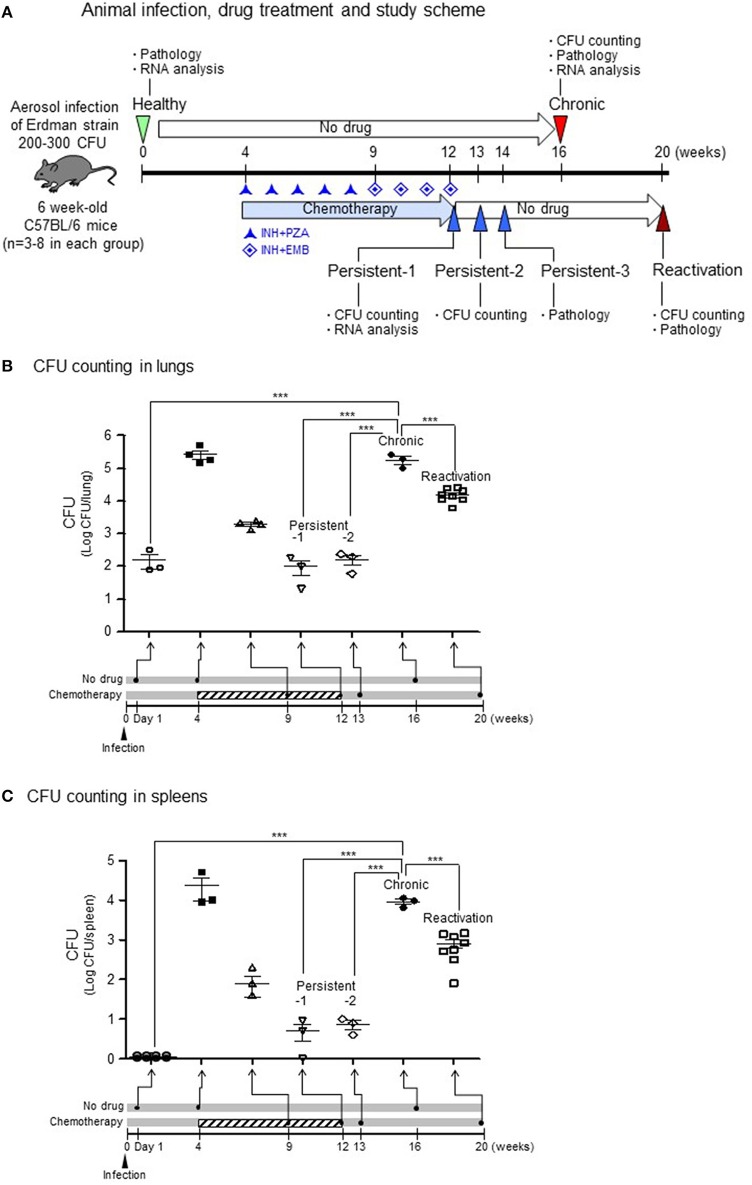
CFU in lungs or spleens after aerosol infection with *M. tuberculosis* bacilli.**(A)** Experimental design for TB infection. For the chronic stage, mice were not treated with any anti-TB agents for 16 weeks after aerosol infection of 200–300 colony-forming units (CFU). For the persistent infection, isoniazid (INH) and pyrazinamide (PZA; from the 4th to 9th weeks) and INH and ethambutol (EMB; from the 9th to 12th weeks) were administrated after aerosol infection. For the reactivation, the persistent stage mice were no longer treated with anti-TB agents (from the 12th to 20th weeks). **(B,C)** The lungs and spleens from each stage were homogenized in PBS, and dilutions were plated on 7H10 agar to enumerate CFUs. *N* = 2–8 in each group. One-way ANOVA was used for statistical analysis. ^***^p < 0.001.

The mouse model for Mtb infection established in our laboratory is very similar to infection in humans in terms of infection route and relapse after treatment, which were confirmed by colony-forming unit (CFU) counts (Figure 1A). To induce a chronic/active infection in mice that is comparable to an active infection in humans, one group of mice were not treated with any anti-TB agents for 16 weeks after aerosol infection with 200–300 CFU of Mtb. To mimic post-chemotherapeutic persistent infection, another group of mice were administered isoniazid (INH) and pyrazinamide (PZA; from the 4th to 9th weeks) followed by INH and ethambutol (EMB; from the 9th to 12th weeks) after aerosol infection. This persistent stage was confirmed by inducing reactivation of bacilli growth without further administration of anti-TB agents (from the 12th to 20th weeks). Upon reactivation, the CFU increased by more than 10^4^ in the lungs (Figure 1B) and 10^3^ in the spleens (Figure 1C) at the 20th week, which mimicked the reactivation of Mtb infection and suggested that the persistent stage in the mice model might be similar to post-chemotherapeutic stage in humans.

For the chronic stage in untreated mice, the CFU reached to more than 10^5^ in the lungs (Figure 1B, *p* < 0.001 compared to healthy group) and more than 10^4^ in the spleens (Figure 1C, *p* < 0.001 compared to healthy group) at 16 weeks after aerosol infection. During induction of the persistent stage, the CFU declined to < 10^3^ in both lungs (Figure 1B) and 10^2^ in the spleens (Figure 1C) at 8 weeks after initiating INH+PZA treatment. Then, during the stable persistent stage, which established after starting of 8 or 9 weeks of the anti-TB drug treatment, the CFU reached to ~10^2^ in both the lungs (Figure 1B, *p* < 0.001 compared to chronic stage) and the spleens (Figure 1C, *p* < 0.001 compared to chronic stage) on persistent stage-1 (1 day) or persistent stage-2 (1 week) after completion of drug treatment.

**Figure 2:**
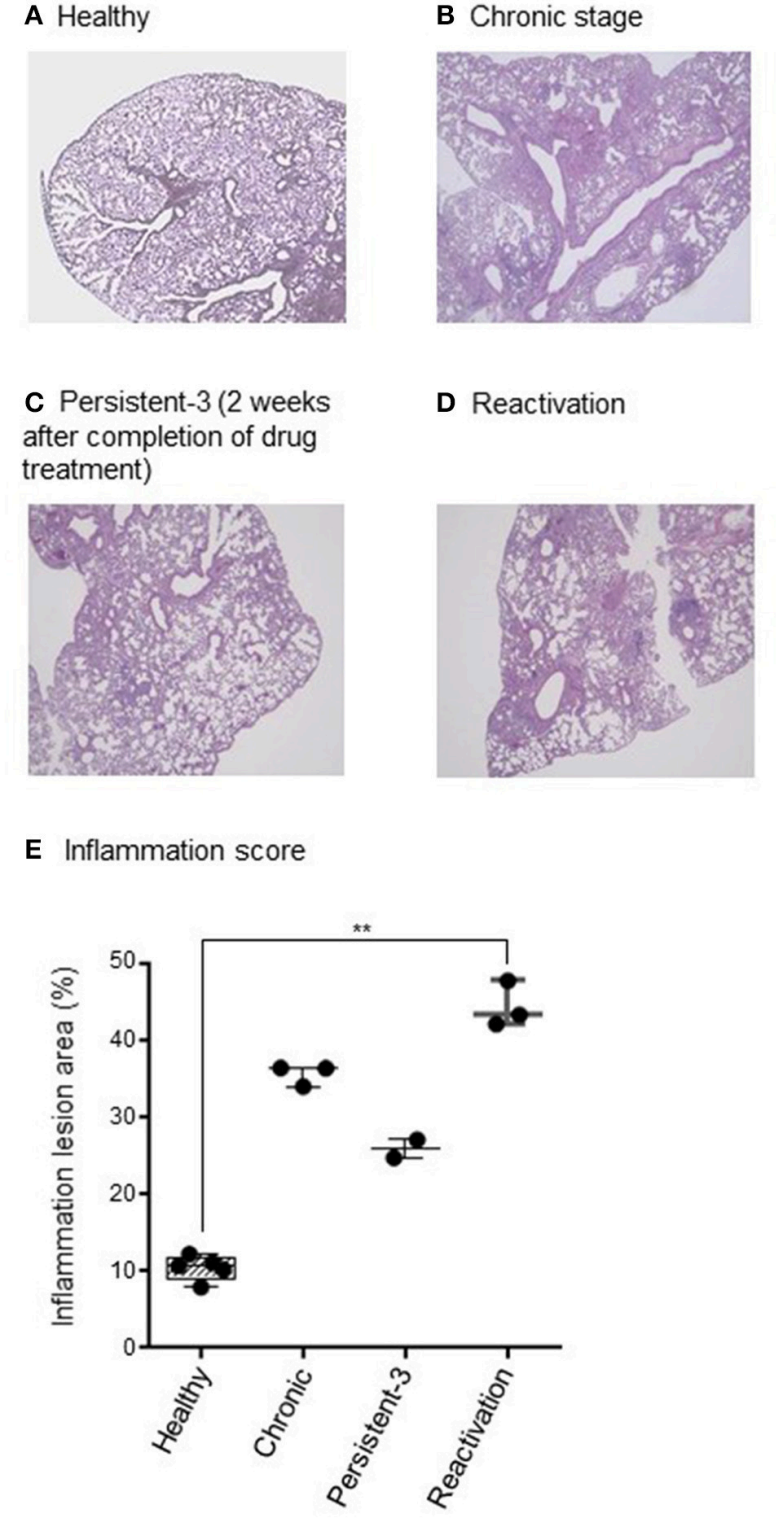
Histopathological findings. **(A–D)** One lung was fixed in phosphate-buffered formaldehyde and embedded in paraffin. Sections were stained with haematoxylin and eosin. Acid-fast *Mycobacterium tuberculosis* bacilli in the lung tissue were detected by Ziehl-Neelsen staining. The stained slides were observed under a light microscope (×40 magnification) and the representative picture are shown. **(E)** Lung inflammation lesions were evaluated by ImageJ software (National Institutes of Health, Bethesda, Maryland, USA). Results were represented as the percentage of area with lesions. *N* = 2–8 in each group. Each dot denotes each individual. One-way ANOVA was used for statistical analysis. ^**^p < 0.01.

Histopathology of chronic stage lung tissues revealed multiple dispersed granulomas, infiltrated mostly with macrophages and neutrophils (Figure 2B) compared with those of healthy group (Figure 2A). At persistent stage-1 (1 day after completion of drug treatment), some pathology of chronic infection was still observed (data not shown), suggesting the tissues had not recovered from the inflammation. The granuloma formation and inflammation were reduced at persistent stage-3, which was 2 weeks after the completion of drug treatment (Figure 2C), suggesting a stable persistent stage had been reached. This persistent stage was supported by multiple granuloma formation of reactivation stage (Figure 2D), which was observed 6 weeks after no anti-TB drug treatment following completion of the INH+PZA or INH+EMB treatment. The percentage of inflammation lesions of each stage showed the similar pattern compared to CFU results. The score for lung inflammation lesions were 8.0–12.2% in healthy group, 34.1–36.5% in chronic group, 24.8–27.1% in persistent group and 42.2–47.9% in reactivation group, respectively (Figure 2E). The difference between healthy and reactivation groups is significant (*p* < 0.01).

### Screening of differentially expressed genes in lung tissues by microarray analysis

**Figure 3:**
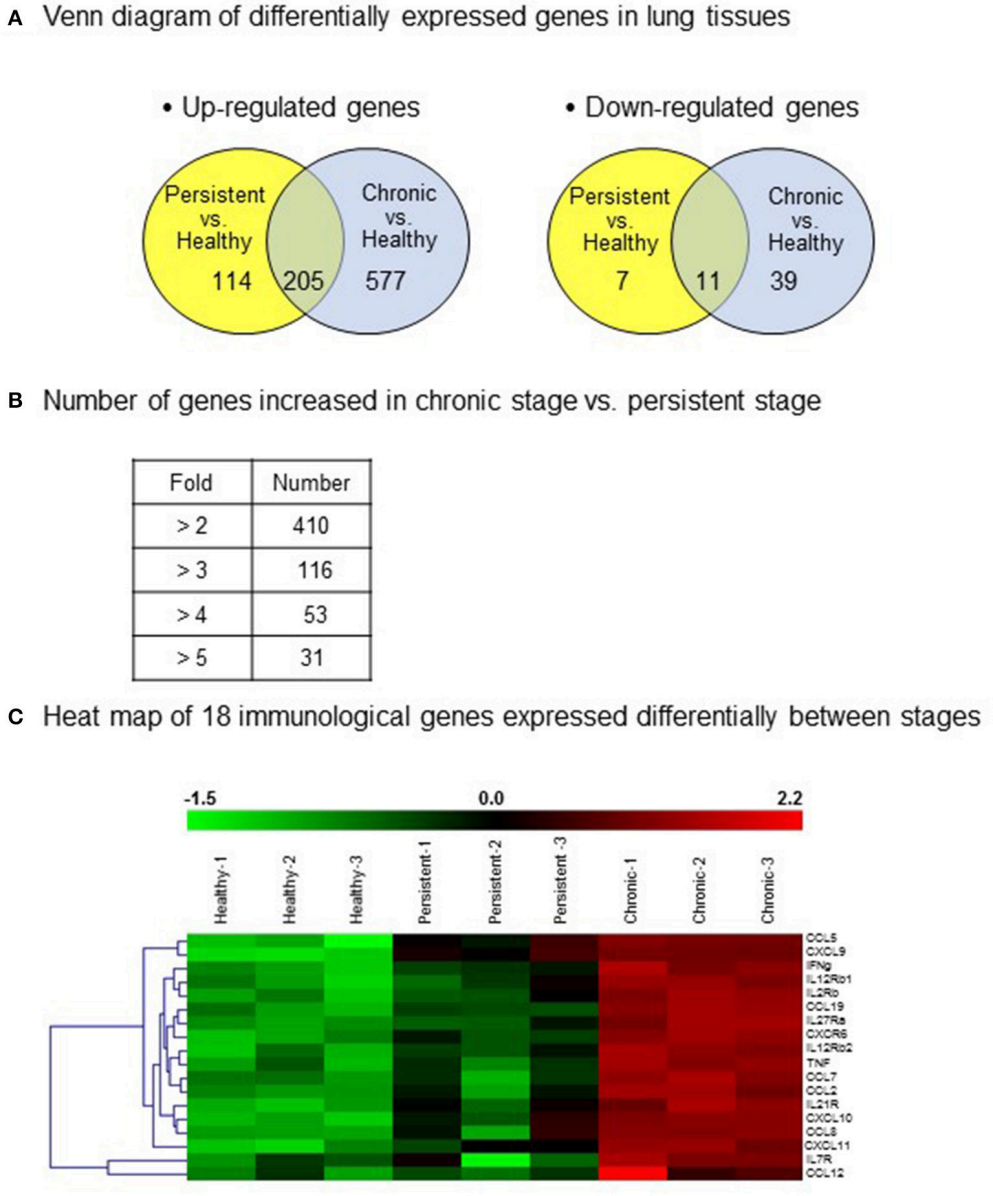
Differentially expressed genes in the various infection stages of tuberculosis. For microarray analysis, a GeneChip® (Affymetrix) containing more than 698,000 total probes and 26,515 RefSeq (Entrez) genes was used. Genes with significantly changed expression values (*p* < 0.05) were selected. **(A)** A Venn diagram of the differentially expressed genes in the lung tissues. Up-regulation >2-fold, and down-regulation <1/2-fold. **(B)** Number of genes with increased expression in the chronic stage vs. the persistent stage. **(C)** Heat map showing the 18 genes chosen for further study.

In an attempt to screen gene expression in lung tissues, a GeneChip® containing more than 698,000 total probes and 26,515 RefSeq (Entrez) genes was used. Compared with that in the healthy group, 577 genes in chronic stage mice and 114 genes in persistent stage mice were found to be up-regulated by more than 2-fold (Figure 3A). Among them, the expression levels of 205 genes were increased in both the persistent and chronic stages. On the other hand, 39 genes in chronic stage mice and 7 genes in persistent stage mice were down-regulated compared with those in the healthy group (Figure 3A). Eleven genes were down-regulated in both the persistent and chronic stages.

Importantly, compared with those in the persistent stage, the expression levels of 410 genes were increased by more than 2-fold, and that of 31 genes by more than 5-fold, in the chronic stage (Figure 3B). The immune response genes that were increased by more than 2-fold are listed. Based on the microarray results, we identified 18 immune response-related genes which were obviously up-regulated during the chronic stage compared with those during the persistent stage (Figure 3C, shown in red in the heat map). Among these 18 genes, we selected 11 cytokine genes for further study. The other seven genes were excluded because they are cytokine receptor genes [viz., interleukin (IL)-12β1 receptor, IL-12β2 receptor, IL-2 receptor, IL-7 receptor, IL-21 receptor, IL-27 receptor, and C-X-C motif chemokine receptor 6 (CXCR6)]. In addition to these immune response genes, the other notably increased genes in the chronic stage were inducible nitric oxide synthase 2, matrix metallopeptidase 12, CD86, CD80, and molecules responsible for T cell activation such as CD3, CD6, lymphocyte-specific protein tyrosine kinase, interleukin-2-inducible T-cell kinase, and linker for activation of T cells (Table 1). The cDNA microarray data are available at NCBI GEO (accession No.: GSE97835).

**Table 1:** Expression of immune response-related genes in the chronic and persistent stages of tuberculosis infection by cDNA microarray analysis.

**Chronic/ persistent**	**Gene symbol**	**Gene description**
5.8	Nos2	nitric oxide synthase 2, inducible
5.2	LOC630751	interferon-inducible GTPase 1-like
5.2	Ly6i	lymphocyte antigen 6 complex, locus I
4.2	Slamf8	signaling lymphocytic activation molecule family member 8
3.6	Tnf	tumor necrosis factor
3.6	Mmp12	matrix metallopeptidase 12
3.5	Cxcl10	chemokine (C-X-C motif) ligand 10
3.4	Cxcr6	chemokine (C-X-C motif) receptor 6
3.2	Ccr5	chemokine (C-C motif) receptor 5
3.1	Ccl8	chemokine (C-C motif) ligand 8
3.0	Xcr1	chemokine (C motif) receptor 1
2.8	Ifng	interferon gamma
2.8	Ccl19	chemokine (C-C motif) ligand 19
2.8	Slamf7	signaling lymphocytic activation molecule family member 7
2.7	Tnfrsf9	tumor necrosis factor receptor superfamily, member 9
2.7	Cd72	CD72 antigen
2.7	Fcgr1	Fc receptor, IgG, high affinity I
2.7	Ccl7	chemokine (C-C motif) ligand 7
2.6	Cxcl9	chemokine (C-X-C motif) ligand 9
2.6	Cd86	CD86 antigen
2.5	Cd3e	CD3 antigen, epsilon polypeptide
2.5	Mmp13	matrix metallopeptidase 13
2.5	Cd4	CD4 antigen
2.5	Ccl2	chemokine (C-C motif) ligand 2
2.4	Msr1	macrophage scavenger receptor 1
2.4	Cd3g	CD3 antigen, gamma polypeptide
2.4	Cd80	CD80 antigen
2.4	Gm2023	predicted gene 2023/ chemokine (C-C motif) ligand 19
2.4	Cd6	CD6 antigen
2.4	Cxcl11	chemokine (C-X-C motif) ligand 11
2.3	Ctss	cathepsin S
2.3	Ccl19	chemokine (C-C motif) ligand 19
2.3	Cd68	CD68 antigen
2.3	Cxcl5	chemokine (C-X-C motif) ligand 5
2.3	Lck	lymphocyte protein tyrosine kinase
2.2	Ly9	lymphocyte antigen 9
2.2	Slamf6	signaling lymphocytic activation molecule family member 6
2.2	Socs1	suppressor of cytokine signaling 1
2.2	Itk	IL2 inducible T cell kinase
2.2	Ccl19	chemokine (C-C motif) ligand 19
2.2	Ccl19	chemokine (C-C motif) ligand 19
2.2	Cd5	CD5 antigen
2.2	Cd40lg	CD40 ligand
2.1	Ccl20	chemokine (C-C motif) ligand 20
2.1	Cd52	CD52 antigen
2.1	Cd8b1	CD8 antigen, beta chain 1
2.1	Il23r	interleukin 23 receptor
2.1	Slamf9	signaling lymphocytic activation molecule family member 9
2.1	Ccl12	chemokine (C-C motif) ligand 12
2.0	Cxcr3	chemokine (C-X-C motif) receptor 3
2.0	Il21r	interleukin 21 receptor
2.0	Lat	linker for activation of T cells

*For microarray analysis, a GeneChip® (Affymetrix) containing more than 698,000 total probes and 26,515 RefSeq (Entrez) genes was used. Among them, genes relevant to immune responses and increased more than 2-fold are shown*.

### Analysis of 11 cytokine genes by cDNA microarray assay and quantitative RT-PCR

**Figure 4:**
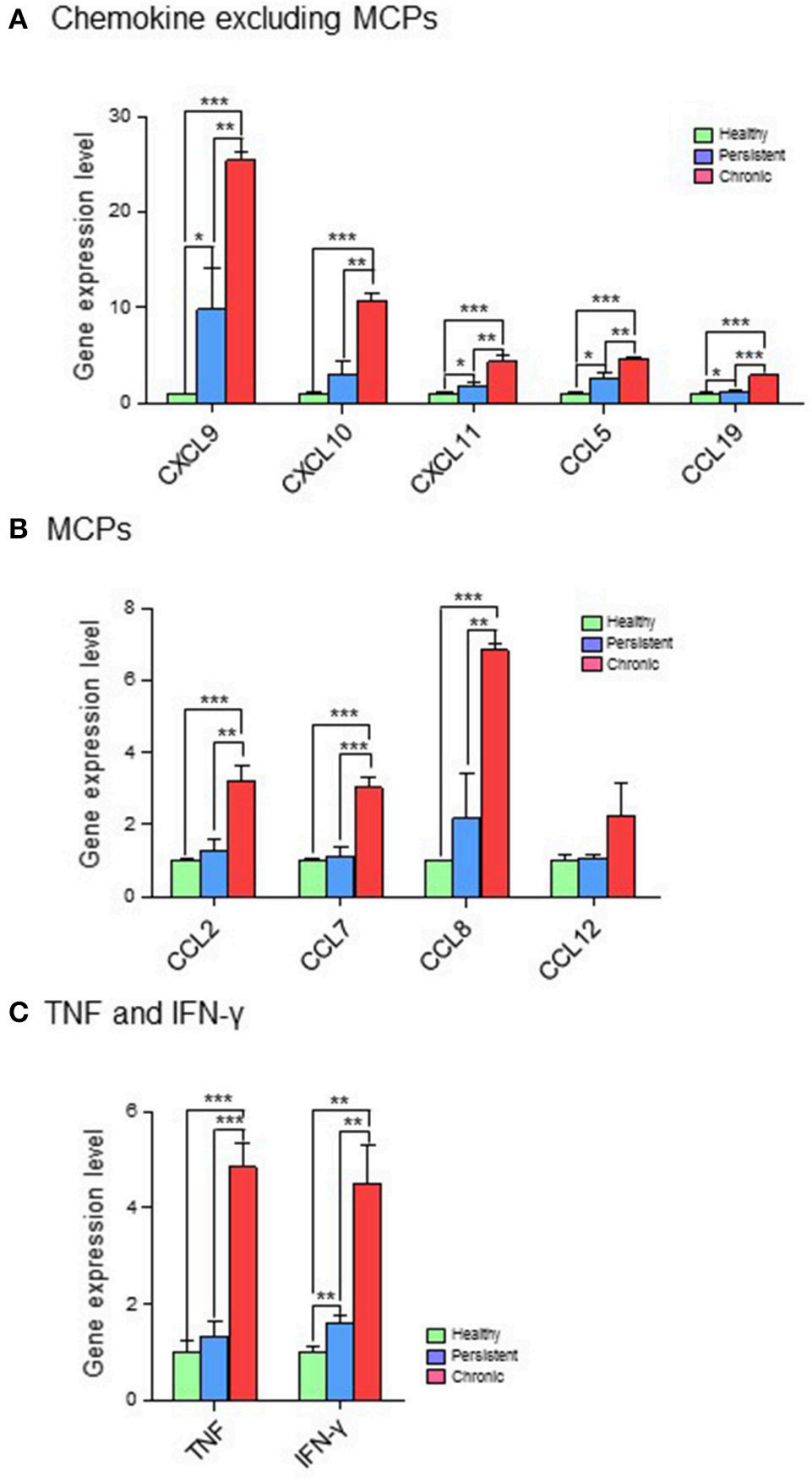
cDNA microarray analysis of 11 genes expressed differentially in the various infection stages of tuberculosis. For microarray analysis, a GeneChip® (Affymetrix) containing more than 698,000 total probes and 26,515 RefSeq (Entrez) genes was used. Genes with expression level changes of 2-fold or greater were selected. **(A)** Chemokines, excluding myocyte chemoattractant proteins (MCPs). **(B)** MCPs. **(C)** Tumor necrosis factor (TNF) and interferon (IFN)-γ. ^*^p < 0.05, ^**^p < 0.01, ^***^p < 0.001.

**Figure 5:**
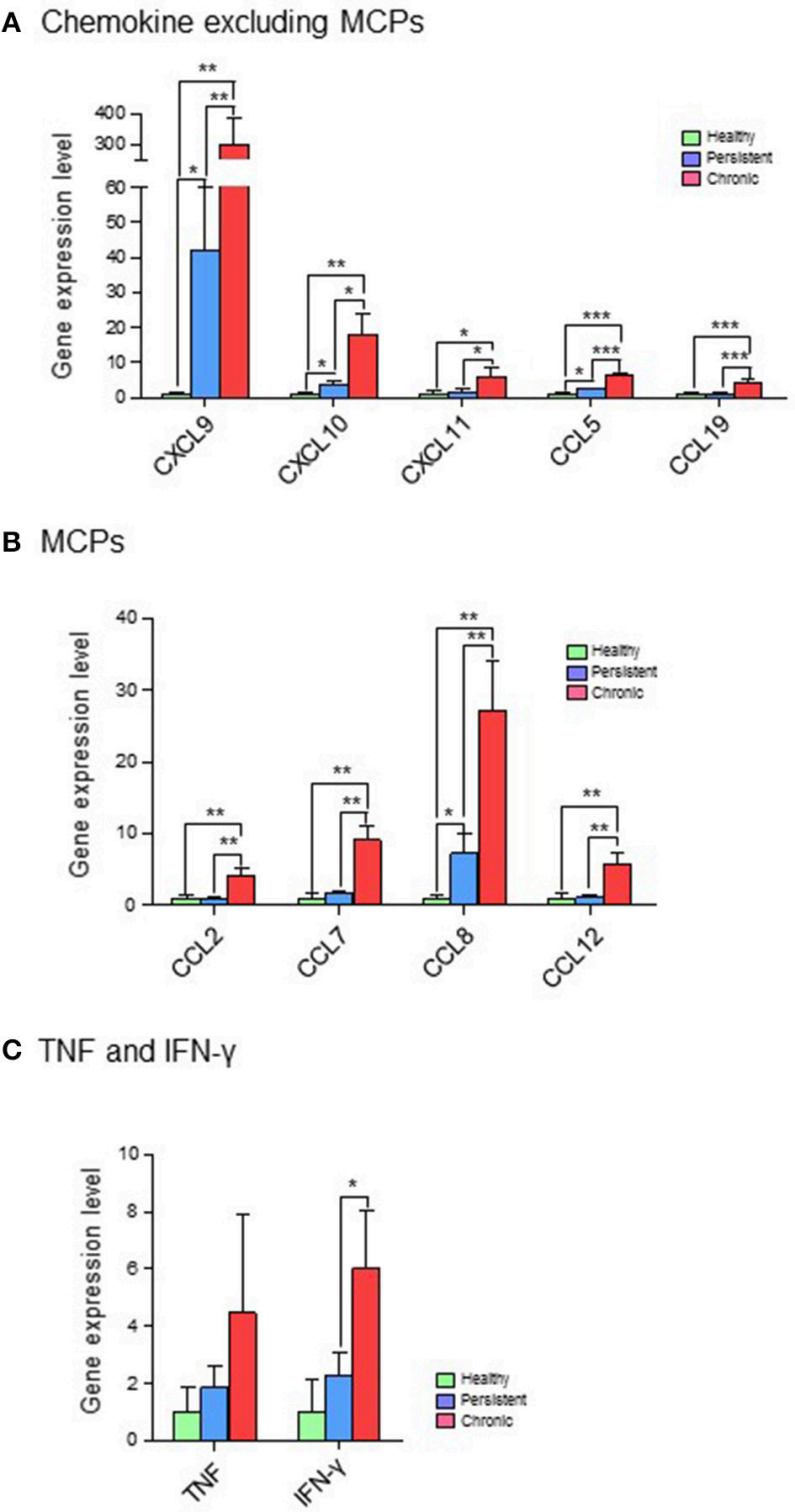
Quantitative RT-PCR analysis of 11 genes expressed differentially in the various infection stages of tuberculosis. Real-time PCR was performed with FastStart Universal Power SYBR Green Master (ROX) (Roche Diagnostics) using the 7,500 Real-Time PCR system (Applied Biosystems). In brief, 1 μL of cDNA was added to a PCR mixture consisting of 12.5 μL of FastStart SYBR green master mix, 11 μL of RNase-free water, and 10 μM of each primer. The PCR protocol was 10 min at 95°C, and 40 cycles of 95°C for 15 s and 60°C for 1 min. StepOne version 2.0.2 software (Applied Biosystems) was used to calculate the expression levels of target genes in the samples, relative to that in the control samples, using the comparative cycle threshold method (ΔΔCT). Expression values for target genes were normalized against that of glyceraldehyde-3-phosphate dehydrogenase (GAPDH). **(A)** Chemokines, excluding myocyte chemoattractant proteins (MCPs). **(B)** MCPs. **(C)** Tumor necrosis factor (TNF) and interferon (IFN)-γ. ^*^p < 0.05, ^**^p < 0.01, ^***^p < 0.001.

Based on their immunological functions, the 11 selected genes were clustered into three groups: (1) chemokines, excluding monocyte chemoattractant proteins (MCPs; CXCL9, CXCL10, CXCL11, CCL5, and CCL19); (2) MCPs (CCL2, CCL7, CCL8, and CCL12); and (3) tumor necrosis factor (TNF) and IFN-γ. The expression levels of all 11 genes were significantly higher in the chronic stage group than in the persistent stage or healthy groups. Although the expression of chemokines such as CXCL9, CXCL10, CXCL11, CCL5, and CCL19 was higher in the persistent stage group than in the healthy group in the cDNA microarray analysis (Figure 4A; Table S2) and quantitative RT-PCR (Figure 5A; Table S2), the difference between the chronic stage vs. the persistent stage or healthy group was more obvious and significant (Tables S1, S3). Among the 11 genes, CXCL9 expression in the chronic stage was notably high, with a 7.2-fold increase over that of the persistent stage (*p* = 0.0067) as shown by quantitative RT-PCR (Table S1). More interestingly, our microarray study showed that the expression of MCPs was higher during the chronic stage than in the persistent stage (Figure 4B; Table S1). The quantitative RT-PCR analysis (Figure 5B; Table S1) showed similar results, with expression increases of 4.24-fold for CCL2 (*p* = 0.0069), 5.16-fold for CCL7 (*p* = 0.0029), 3.78-fold for CCL8 (*p* = 0.0094), and 5.25-fold for CCL12 (*p* = 0.0081). Among them, CCL8 expression was significantly higher in the persistent stage mice than in the healthy group (7.21-fold, *p* = 0.0179), whereas the CCL2 (0.99-fold, *p* = 0.4698), CCL7 (1.789-fold, *p* = 0.1474), and CCL12 (1.11-fold, *p* = 0.8058) expression levels did not differ significantly between these two mouse groups. Therefore, we suggest the MCPs to be further candidate biomarkers to distinguish each infection stage efficiently. Although, mRNA of TNF-α and IFN-γ (two well-known cytokine indicators for TB) were increased in lung tissues in the microarray analysis (Figure 4C; Table S1), we could not obtain significant results by quantitative RT-PCR (Figure 5C; Table S1).

## Discussion

Although, biomarkers to detect Mtb infection or to distinguish disease stages have been studied for several decades, improvements have not been made successfully. Ideal biomarkers for TB should be key biological factors expressed differentially among the infection stages and specific to the disease. IFN-γ, the indicator used in the IGRA, has been considered a potential biomarker for identifying the presence or absence of Mtb infection and its infection stages (Greveson et al., 2013; Targowski et al., 2014; Weiner and Kaufmann, 2014). Although IGRA, which is more specific and sensitive than the tuberculin skin test, is currently the most commonly used assay to diagnose latent TB infection (Cho et al., 2016), it cannot distinguish latent infection from active TB (Metcalfe et al., 2011). Therefore, to more accurately distinguish between these two stages, the selective combination of immune response genes has been under study. Among them, the protein levels of CXCL10 (IP-10; Tonby et al., 2015; Wergeland et al., 2015; Xiong et al., 2016; regulated under IFN-γ) and levels of mRNA Kim et al., 2015 or protein (Jeong et al., 2015; Lee et al., 2015a) for CXCL9, CXCL10, and CXCL11 have been pointed out by other researchers. These studies were mostly performed with blood mononuclear cells after stimulation with Mtb-specific antigens. Only several papers tested plasma levels without antigen stimulation. One study suggested the possibility of using IFN-γ, IL-4, CCL4, CCL7, and CXCL10 from unstimulated plasma samples to discriminate active TB from latently infected contacts and to monitor anti-TB treatment (Mihret et al., 2013). Another study suggested CXCR3 ligands (CXCL9 and CXCL10) as useful surrogate markers in plasma for diagnosing active TB clinically (Lee et al., 2015a). More recently, when plasma levels of IL-2, IL-4, IL-6, IL-10, TNF-α, and IFN-γ were determined during the follow-up of anti-TB chemotherapy, slow responders showed significantly higher IL-2 and IL-4 levels at baseline than did fast responders, effectively differentiating the two responder types (Iqbal et al., 2016). Considering these reports, a systematic analysis of immune response genes is essential to understand diverse immune reactions in tuberculosis.

In this study, we adopted a mouse model for Mtb infection, which had been established in our laboratory based on the Cornell model infection with the Mtb (Ha et al., 2003). The expression of cytokines and other immune response-related genes was evaluated in lung tissues from mice in the post-chemotherapeutic persistent stage and in the chronic stage (comparable to active infection in humans), as well as in healthy mice. To evaluate the *in situ* immune responses, RNA was purified directly from the lung tissues without any further antigen stimulation *in vitro*, and cDNA microarray and quantitative RT-PCR analyses were performed.

First, our data showed that both mRNA levels of IFN-γ and CXCL10 were increased during the chronic stage compared with the persistent stage. IFN-γ and CXCL10 (Holm et al., 2014; Latorre et al., 2014; Jeong et al., 2015; Tonby et al., 2015; Wergeland et al., 2015) are already well-known major biomarkers for TB. Our results are in agreement with those of previous studies, and are not surprising given that CXCL10 is known to be under the regulation of IFN-γ. However, our study also showed that the mRNA expression of CXCL10 was much higher than that of IFN-γ; therefore, CXCL10 may be easier to detect. Furthermore, because the changes in CXCL10 levels were more significant than those in IFN-γ levels (Figures 4A,C, 5A,C), CXCL10 could be a more useful biomarker to screen the infection stages of TB.

In addition to IFN-γ and CXCL10, our study suggested that mRNA levels of chemokines such as CXCL9, CXCL11, CCL5, and CCL19 were increased distinctly in the chronic stage relative to the persistent stage (Figures 4A, 5A). Similar to our results, the plasma levels of CXCL9, CXCL10, and CXCL11 were reported in another study to be higher in active TB patients than in healthy persons (Lee et al., 2015a), whereas the IFN-γ levels did not differ in that paper. A recent study also showed that protein levels of CXCR3 ligands, such as CXCL9 and CXCL11, were higher in sera from TB patients than in sera from healthy persons (Lee et al., 2015a). However, there has been no report about CCL5 and CCL19 until this present work.

In our study, although the net mRNA expression levels of CCL5 and CCL19 were lower than that of CXCL9 or CXCL11, the significant differences between the chronic stage and the persistent or healthy stages suggest these ligands as biomarker candidates. One recent paper assessing blood mononuclear cells from tuberculosis patients suggests that the polymorphism (TT genotype) of CCL5 may play an important role to decrease CCL5 expression in T cells to interfere immunity against tuberculosis (Singh et al., 2017). Interestingly, among selected chemokines, CXCL9 distinguished the persistent stage from healthy group notably (41.85-fold).

Besides IFN-γ, CXCL10, and CXCL9, which have been intensively studied by other researchers, our study revealed that mRNA levels of MCPs such as CCL2, CCL7, CCL8, and CCL12 were increased in lung tissues. MCPs induce the recruitment of multiple subsets of leukocytes, including monocytes. A few studies have reported CCL2 (Mihret et al., 2013) and CCL7 (Mihret et al., 2013) to be increased in sera of patients with active TB, which is highly comparable to our results in the lungs (Figures 4B, 5B). Recently, genetic polymorphism of CCL2 has been suggested to be associated with the susceptibility to TB (Nonghanphithak et al., 2016). Another study has suggested the association of CCL2 with the pathogenesis of TB, by showing that the 6 kDa early secretory antigenic target (ESAT-6) of Mtb-induced CCL2 production in mouse bone marrow-derived macrophages and that the inhibition of p38 enhanced ESAT-6-induced CCL2 production (Ma et al., 2016). Therefore, the increase of CCL2 and CCL7 in our mouse Mtb infection model is also applicable for the understanding of the pathogenesis of human TB. Another important piece of information in our study is the increased expression of CCL12 in lung tissues of the chronic stage (Figures 4B, 5B). The involvement of CCL12 in TB has not been reported before.

Taking the results together, through screening of ~30,000 genes using *in situ* infected lung tissues, we have identified several TB biomarkers which might be suitable for distinguishing the chronic/active stage from the post-chemotherapeutic persistent stage. Our study showed that the levels of MCPs in lung tissues could distinguish between the two stages significantly. We are the first to observe significant up-regulation of CCL5, CCL19, and CCL12 in the chronic stage of TB. Furthermore, the MCPs including CCL2, CCL7, CCL8, and CCL12, which were evaluated in a few studies, might be useful biomarkers to distinguish the infection stages of this disease. Quantitative RT-PCR showed the expression levels of all four MCPs to be significantly higher in the chronic stage than in the persistent stage, whereas only CCL8 distinguished the persistent stage (7.21-fold) from the healthy group. CCL2, CCL7, and CCL12 could not distinguish the persistent stage mice from the healthy group.

Research on TB diagnosis is progressing to the point of finding markers in sputum samples (Marais and Pai, 2007; Sia and Wieland, 2011). The advantage of the sputum-based test is that whereas routine clinical samples are obtained from relatively inaccessible sites (Lee, 2015), sputum can be easily monitored (Jacobs et al., 2016). Moreover, the trend is to omit the process of antigen stimulation, because diagnosis using Mtb antigen-induced cytokines/chemokines by blood mononuclear cells is considered inconvenient, labor intensive, and time consuming (Yang et al., 2014). Therefore, we are certain that our study covering multiple gene arrays will provide abundant information regarding the comparison of tissues and sera.

In conclusion, we have identified several new biomarkers that may be applied for TB screening and for the diagnosis of chronic/active TB or post-chemotherapeutic persistent stage. Of particular importance, we suggest evidence that the combined use of multiple indicators may raise the sensitivity and specificity of TB detection.

## Ethics statement

All animal experiments were performed in compliance with the Yonsei University Institutional Animal Care guidelines, and protocols were reviewed and approved by the Institutional Animal Care and Use Committees of the Laboratory Animal Research Center at Yonsei University Health System (Permit Number: 2014-0301).

## Author contributions

SC and IC designed research. SP, SB, and YJ performed experiments and analyzed the data. IC wrote the manuscript. All authors reviewed the final version of the manuscript.

## Acknowledgment

This research was supported by a grant of the Korea Health Technology R&D Project through the Korea Health Industry Development Institute (KHIDI), funded by the Ministry of Health and Welfare, Republic of Korea (Grant number: HI13C0847).

### Conflict of interest statement

The authors declare that the research was conducted in the absence of any commercial or financial relationships that could be construed as a potential conflict of interest.
