# Supplementary material for: Unique Chemokine Profiles of Lung Tissues Distinguish Post-chemotherapeutic Persistent and Chronic Tuberculosis in a Mouse Model
Source: Front Cell Infect Microbiol. 2017 Jul 13;7:314. doi: 10.3389/fcimb.2017.00314 (PMC5508001; doi:10.3389/fcimb.2017.00314)
Supplement: Supplementary file 1 [file DataSheet1.PDF]

Supplementary Information

Table S1. Statistical analysis of data obtained by cDNA microarray and quantitative RT-PCR analyses (chronic vs. persistent)

Gene		Microarrays		Quantitative RT-PCR	
		Fold change	P-value	Fold change	P-value
Chemokines excluding MCPs	CXCL9	2.59	0.0035	7.20	0.0067
	CXCL10	3.55	0.0011	4.84	0.0162
	CXCL11	2.37	0.0022	3.91	0.0464
	CCL5	1.81	0.0052	2.72	0.0008
	CCL19	2.32	<0.0001	4.16	0.0008
MCPs	CCL2	2.47	0.0031	4.24	0.0069
	CCL7	2.66	0.0010	5.16	0.0029
	CCL8	3.14	0.0029	3.78	0.0094
	CCL12	2.06	0.1064	5.25	0.0081
TNF and IFN--γ	TNF	3.61	0.0004	2.41	0.2679
	IFN-γ	2.80	0.0032	2.63	0.0405

For microarray analysis, a GeneChip® (Affymetrix) containing more than 698,000 total probes and 26,515 RefSeq (Entrez) genes was used. Quantitative real-time PCR was performed with FastStart Universal Power SYBR Green Master (ROX) (Roche Diagnostics) using the 7500 Real-Time PCR system (Applied Biosystems). The results of microarray and quantitative RT-PCR were analysed by Student's unpaired *t*-test (two-tailed).

Table S2. Statistical analysis of data obtained by cDNA microarray and quantitative RT-PCR analyses (persistent vs. healthy)

Gene		Microarrays		Quantitative RT-PCR	
		Fold change	P-value	Fold change	P-value
Chemokines excluding MCPs	CXCL9	9.83	0.0236	41.85	0.0176
	CXCL10	3.04	0.0693	3.70	0.0189
	CXCL11	1.85	0.0102	1.56	0.4692
	CCL5	2.55	0.0131	2.29	0.0420
	CCL19	1.26	0.0146	1.07	0.7142
MCPs	CCL2	1.30	0.1675	0.99	0.4698
	CCL7	1.14	0.4095	1.78	0.1474
	CCL8	2.18	0.1727	7.21	0.0179
	CCL12	1.08	0.4760	1.11	0.8058
TNF and IFN--γ	TNF	1.35	0.1865	1.85	0.2775
	IFN-γ	1.61	0.0054	2.29	0.2260

For microarray analysis, a GeneChip® (Affymetrix) containing more than 698,000 total probes and 26,515 RefSeq (Entrez) genes was used. Quantitative real-time PCR was performed with FastStart Universal Power SYBR Green Master (ROX) (Roche Diagnostics) using the 7500 Real-Time PCR system (Applied Biosystems). The results of microarray and quantitative RT-PCR were analysed by Student's unpaired *t*-test (two-tailed).

Table S3. Statistical analysis of data obtained by cDNA microarray and quantitative RT-PCR analyses (chronic vs. healthy)

Gene		Microarrays		Quantitative RT-PCR	
		Fold change	P-value	Fold change	P-value
Chemokines excluding MCPs	CXCL9	25.44	<0.0001	301.26	0.0036
	CXCL10	10.78	<0.0001	17.90	0.0084
	CXCL11	4.38	0.0006	6.10	0.0328
	CCL5	4.62	<0.0001	6.23	0.0006
	CCL19	2.92	<0.0001	4.46	0.0007
MCPs	CCL2	3.22	0.0008	4.19	0.0078
	CCL7	3.03	0.0003	9.17	0.0024
	CCL8	6.83	<0.0001	27.26	0.0028
	CCL12	2.22	0.0929	5.83	0.0089
TNF and IFN--γ	TNF	4.86	0.0003	4.46	0.1649
	IFN-γ	4.52	0.0016	6.02	0.0533

For microarray analysis, a GeneChip® (Affymetrix) containing more than 698,000 total probes and 26,515 RefSeq (Entrez) genes was used. Quantitative real-time PCR was performed with FastStart Universal Power SYBR Green Master (ROX) (Roche Diagnostics) using the 7500 Real-Time PCR system (Applied Biosystems). The results of microarray and quantitative RT-PCR were analysed by Student's unpaired *t*-test (two-tailed).

Table S4. Primer sequences for quantitative RT-PCR

Gene description	Gene symbol	Primer sequence (5'-3')<br>Forward & Reverse	Sequence accession number
Chemokine (C-X-C motif) ligand 9	CXCL9	AGT CCG CTG TTC TTT TCC TC<br>TGA GGT CTT TGA GGG ATT TGT AG	NM_008599
Chemokine (C-X-C motif) ligand 10	CXCL10	TCA GCA CCA TGA ACC CAA G<br>CTA TGG CCC TCA TTC TCA CTG	NM_021274
Chemokine (C-X-C motif) ligand 11	CXCL11	ATG GCA GAG ATC GAG AAA GC<br>TGC ATT ATG AGG CGA GCT TG	NM_019494
Chemokine (C-C motif) ligand 5	CCL5	GGG TAC CAT GAA GAT CTC TGC<br>TCT AGG GAG AGG TAG GCA AAG	NM_013653
Chemokine (C-C motif) ligand 19	CCL19	AGA CTG CTG CCT GTC TGT GA<br>GCC TTT GTT CTT GGC AGA AG	NM_011888
Chemokine (C-C motif) ligand 2	CCL2	CAT CAG TCC TCA GGT ATT GGC<br>TTG TGA TTC TCC TGT AGC TCT TC	NM_011333
Chemokine (C-C motif) ligand 7	CCL7	TCT CTC ACT CTC TTT CTC CAC C<br>GGG ATC TTT TGT TTC TTG ACA TAG C	NM_013654
Chemokine (C-C motif) ligand 8	CCL8	ACA ATA TCC AGT GCC CCA TG<br>CAT GTA CTC ACT GAC CCA CTT C	NM_021443
Chemokine (C-C motif) ligand 12	CCL12	CAT CAG TCC TCA GGT ATT GGC<br>TTG TGA TTC TCC TGT AGC TCT TC	NM_011331
Tumor necrosis factor	TNF	AGG GAT GAG AAG TTC CCA AAT G<br>GGC TTG TCA CTC GAA TTT TGA GA	NM_001278601
Interferon gamma	IFN-γ	TTT AAC TCA AGT GGC ATA GAT GTG G<br>TGC AGG ATT TTC ATG TCA CCA T	NM_008337
